# Lower Levels of GABAergic Function Markers in Corticotropin-Releasing Hormone-Expressing Neurons in the sgACC of Human Subjects With Depression

**DOI:** 10.3389/fpsyt.2022.827972

**Published:** 2022-02-25

**Authors:** Hyunjung Oh, Dwight Newton, David Lewis, Etienne Sibille

**Affiliations:** ^1^Campbell Family Mental Health Research Institute of CAMH, Toronto, ON, Canada; ^2^Departments of Pharmacology and Toxicology, University of Toronto, Toronto, ON, Canada; ^3^Department of Psychiatry, University of Pittsburgh, Pittsburgh, PA, United States; ^4^Department of Psychiatry, University of Toronto, Toronto, ON, Canada

**Keywords:** MDD (major depressive disorder), CRH, cell dysfunction, GABA, sgACC, human post mortem tissue

## Abstract

**Rationale:**

A previous transcriptome meta-analysis revealed significantly lower levels of corticotropin-releasing hormone (CRH) mRNA in corticolimbic brain regions in major depressive disorder (MDD) subjects, suggesting that cortical CRH-expressing (CRH+) cells are affected in MDD. Rodent studies show that cortical CRH is mostly expressed in GABAergic interneurons; however, the characteristic features of CRH+ cells in human brain cortex and their association with MDD are largely unknown.

**Methods:**

Subgenual anterior cingulate cortex (sgACC) of human subjects without brain disorders were labeled using fluorescent *in situ* hybridization (FISH) for CRH and markers of excitatory (SLC17A7), inhibitory (GAD1) neurons, as well as markers of other interneuron subpopulations (PVALB, SST, VIP). MDD-associated changes in CRH+ cell density and cellular CRH expression (*n* = 6/group) were analyzed. RNA-sequencing was performed on sgACC CRH+ interneurons from comparison and MDD subjects (*n* = 6/group), and analyzed for group differences. The effect of reduced BDNF on CRH expression was tested in mice with blocked TrkB function.

**Results:**

About 80% of CRH+ cells were GABAergic and 17.5% were glutamatergic. CRH+ GABAergic interneurons co-expressed VIP (52%), SST (7%), or PVALB (7%). MDD subjects displayed lower CRH mRNA levels in GABAergic interneurons relative to comparison subjects without changes in cell density. CRH+ interneurons show transcriptomic profile suggesting lower excitability and less GABA release and reuptake. Further analyses suggested that these molecular changes are not mediated by altered glucocorticoid feedback and potentially occur downstream for a common modulator of neurotrophic function.

**Summary:**

CRH+ cells in human sgACC are a heterogeneous population of GABAergic interneurons, although largely co-expressing VIP. Our data suggest that MDD is associated with reduced markers of inhibitory function in sgACC CRH+ interneurons, and provide further evidence for impaired GABAergic function in the cortex in MDD.

## Introduction

Major depressive disorder (MDD) brings a huge burden to our society due to its high prevalence, mortality, and recurrence rates. It is estimated that the lifetime prevalence of MDD is 15–18% (1) with over 300 million people living with depression globally (2). The majority of suicide victims have MDD (3) and about 80% of MDD patients experience recurrent episodes throughout their lifetime (4, 5). Selective serotonin reuptake inhibitors (SSRIs) are the first-line treatment for MDD, yet they can cause debilitating side effects such as nausea, weight gain, sexual dysfunction, and suicidal ideation (6). Moreover, SSRIs take weeks to show therapeutic effects. Remission rates following 14 weeks of SSRI treatment are only 30% (7), and 20–35% of patients develop a chronic illness during a 4-year follow-up (8). These low treatment efficacy and high relapse rates emphasize the need for a better understanding of the pathophysiology of MDD and the development of more advanced treatments.

Corticotropin-releasing hormone (CRH) is significantly downregulated in corticolimbic regions of MDD patients in our prior meta-analysis of altered gene expression in MDD, suggesting that cortical CRH-expressing (CRH+) cells are affected in MDD (9). CRH is a neuropeptide better known for its role in regulating the hypothalamus/pituitary/adrenal (HPA) stress axis. Indeed, hypothalamic CRH plays a pivotal role in coordinating physiological responses to stress and is implicated in various psychiatric illnesses. CRH is also expressed in extra-hypothalamic regions, mainly in GABAergic interneurons in the neocortex of rodents (10–12). CRH+ neurons demonstrated a great variability in density, laminar distribution and morphology across the monkey neocortex (13). CRH+ neurons and released CRH peptide were shown to modulate cognitive- and mood-related behaviors (14, 15). The association of CRH+ neurons with MDD is also implied by a subsequent RNA sequencing (RNAseq) study performed in the subgenual anterior cingulate cortex (sgACC) of MDD patients (16). In that study, we performed cell-type deconvolution of bulk tissue RNAseq data, which suggested that CRH-expressing (CRH+) neurons are affected by the disease and their transcriptomic changes are synchronized with the episode and remission phases of the illness. Using a probabilistic Bayesian network analysis, we found that these gene expression changes may play a causal role on the disease trajectory. Together, these unbiased, large-scale studies point out that CRH is one of very few genes that can serve as a molecular signature of MDD, which is notorious for its clinical heterogeneity, and that reduced function of cortical CRH+ inhibitory interneurons may contribute to the well-characterized GABAergic pathophysiology of the illness (9, 17–24).

In the current study, we first characterized the cellular identities of CRH+ neurons in human sgACC and investigated potential CRH+ neuron subtype specificity affected in MDD. Using laser-capture microdissection (LCM) and RNAseq techniques, we next assessed potential biological functions affected in CRH+ GABAergic interneurons in a subset of MDD subjects with characterized reduced CRH expression. We predicted that CRH+ neurons are mostly GABAergic and that MDD is associated with dysfunction of CRH+ interneurons.

## Materials and Methods

### Human Postmortem Subjects

Brain samples were obtained during autopsies conducted at the Allegheny County Medical Examiner's Office (Pittsburgh, PA, USA) after consent from next-of-kin and using procedures approved by the University of Pittsburgh's Institutional Review Board and Committee for Oversight of Research and Clinical Training Involving Decedents. Using the results of structured interviews with family members and review of clinical records, an independent committee of experienced research clinicians made consensus DSM-IV diagnoses for each subject (25). To control for biological variance, each MDD subject was paired with a non-psychiatric control subject, matched for sex and as closely as possible for age, postmortem interval and brain pH. Two cohorts were generated for this study. First, five control subjects were investigated for a cell marker colocalization study. Second, six matched pairs of male MDD and comparison subjects were selected based on availability of tissue from a previous cohort designed to investigate disease-associated GABAergic and neurotrophic gene expression changes (22, 24, 26). These samples were used to characterize the cellular and laminar specificity of CRH reduction in MDD. There were no significant group differences on any demographic or technical parameters (Tables 1, 2).

**Table 1:** Demographics of postmortem human subjects used for CRH+ cell characterization.

**Case**	**Age**	**PMI**	**pH**	**RNA ratio**	**RIN**
551	61	16.4	6.6	1.3	8.3
604	39	19.3	7.1	2.1	8.6
795	68	12	6.6	1.6	8.2
857	48	16.6	6.7	2	8.9
1,122	55	15.4	6.7	1.4	7.9

*PMI, postmortem interval; RIN, RNA integrity number*.

**Table 2:** Demographics of postmortem human subjects used for MDD-related RNA expression changes in CRH+ cells.

**Control**						**Major depressive disorder**									
**Case**	**Age**	**PMI**	**pH**	**RNA ratio**	**RIN**	**Case**	**Age**	**PMI**	**pH**	**RNA ratio**	**RIN**	**Suicide**	**Remission**	**Antidepressant**	**Recurrent episode**
1,086	51	24.2	6.8	1.4	8.1	863	51	28.3	7.2	1.5	8.4	N	N	N	N
852	54	8	6.8	1.8	9.1	1,001	53	7.3	6.6	1.4	7.6	N	Full	N	N
1,031	53	23.2	6.8	1.5	8.2	809	50	20	6.9	1.5	8.5	N	Full	Y	N
1,047	43	13.8	6.6	1.8	9	943	56	15.4	6.6	1.5	8.2	Y	Partial	N	Y
789	22	20.1	7	2	7.8	513	24	13.1	6.9	1.5	7	Y	N	N	U
615	62	7.2	6.4	1.4	7.8	600	63	9.9	6.7	1.7	7.1	Y	N	N	N
Mean	47.5	16.1	6.7	1.6	8.3	Mean	49.5	15.7	6.8	1.5	7.8				
SEM	5.7	3.1	0.1	0.1	0.2	SEM	5.4	3.1	0.1	0	0.3				
*p*-value	0.43	0.82	0.42	0.46	0.12										

### Animals and Drug Treatment

NTRK2^F616A^ heterozygote mice, which contain a point mutation in the ATP binding domain of NTRK2 that can be selectively blocked by ATP competitive kinase inhibitor 1NMPP1 (27), were purchased from Jackson laboratories (Bar Harbor, ME, USA) and intercrossed to generate homozygote mice. To temporarily control NTRK2-mediated signaling, male homozygote mice (9–10 weeks old) were fed with 25 μM 1NMPP1 (MilliporeSigma, Burlington, MA, USA; catalog no. 529581) or vehicle (0.0003% DMSO) *via* drinking water for 3 weeks and sacrificed, as described in a previous study (28).

### RNA Extraction and Quantitative PCR (qPCR)

Human sgACC samples containing all cortical layers of gray matter were harvested from coronal sections and processed as previously described (29). In brief, total RNA was isolated from tissue sections stored in Trizol and further purified with RNeasy columns from RNeasy micro kit (Qiagen, Valencia, CA, USA; catalog no. 74004). cDNA was synthesized by mixing 1 μg of total RNA with oligo-dT primers and SuperScript II Reverse Transcriptase following manufacturer's protocol (Invitrogen, Carlsbad, CA, USA; catalog no. 18064071). Mice anterior cingulate cortices were isolated using micropunch techniques and processed for RNA extraction with Qiagen RNeasy micro kit. Hundred nanogram of total RNA was mixed with qScript cDNA supermix (Quanta BioSciences, Gaithersburg, MD, USA; catalog no. 95047-100) to generate cDNA. PCR products were amplified in triplicate on a CFX Real-Time PCR Detection System (Bio-rad, Hercules, CA, USA), using universal PCR conditions. Results were calculated as the geometric mean of relative intensities compared to three validated internal controls (actin, glyceraldehyde-3- phosphate dehydrogenase and cyclophilin A). CRH primers were purchased from Bio-rad: human CRH primer (catalog no. qHsaCED004537), mouse CRH primer (catalog no. qMmuCED0001593). Primers for three internal controls were designed using Primer 3 plus (http://www.bioinformatics.nl/cgi-bin/primer3plus/primer3plus.cgi) and sequences are provided in Table 3.

**Table 3:** Sequences of primers used in qPCR experiments.

**Species**	**Gene**	**Forward primer sequence**	**Reverse primer sequence**	**Product**
Mouse	Actin (ACTB)	CCTAGCACCATGAAGATCAA	GGAAGGTGGACAGTGAGG	CCTAGCACCATGAAGATCAAGATCATTGCTCCTCCTGAGCGCAAGTACTCTGTGTGGATCGGTGGCTCCATCCTGGCCTCACTGTCCACCTTCC
	Glyceraldehyde 3-phosphate dehydrogenase (GAPDH)	AACTCCCACTCTTCCACCT	CACCACCCTGTTGCTGTA	AACTCCCACTCTTCCACCTTCGATGCCGGGGCTGGCATTGCTCTCAATGACAACTTTGTCAAGCTCATTTCCTGGTATGACAATGAATACGGCTACAGCAACAGGGTGGTG
	Cyclophilin A (PPIA)	CAAACACAAACGGTTCCCAG	TTCACCTTCCCAAAGACCAC	CAAACACAAACGGTTCCCAGTTTTTTATCTGCACTGCCAAGACTGAATGGCTGGATGGCAAGCATGTGGTCTTTGGGAAGGTGAA
Human	Actin (ACTB)	GCATGGGTCAGAAGGATT	GGTACTTCAGGGTGAGGATG	GCATGGGTCAGAAGGATTCCTATGTGGGCGACGAGGCCCAGAGCAAGAGAGGCATCCTCACCCTGAAGTACC
	Glyceraldehyde 3-phosphate dehydrogenase (GAPDH)	AACGAATTTGGCTACAGCA	GGGTCTCTCTCTTCCTCTTGT	AACGAATTTGGCTACAGCAACAGGGTGGTGGACCTCATGGCCCACATGGCCTCCAAGGAGTAAGACCCCTGGACCACCAGCCCCAGCAAGAGCACAAGAGGAAGAGAGAGACCC
	Cyclophilin A (PPIA)	GCAGACAAGGTCCCAAAG	GAAGTCACCACCCTGACAC	GCAGACAAGGTCCCAAAGACAGCAGAAAATTTTCGTGCTCTGAGCACTGGAGAGAAAGGATTTGGTTATAAGGGTTCCTGCTTTCACAGAATTATTCCAGGGTTTATGTGTCAGGGTGGTGACTTC

### Fluorescent *in situ* Hybridization (FISH)

FISH was performed using RNAscope FISH technology (Advanced Cell Diagnostics, Newark, CA, USA; catalog no. 320850) as previously described (30). Briefly, 14 μm-thick fresh-frozen human sgACC sections were thaw-mounted onto Superfrost Plus slides (Thermo Fisher Scientific, Waltham, MA, USA; catalog no. 1255015) and stored at −80°C. On the day of experiment, slides were taken from −80°C and immediately immersed in chilled 4% paraformaldehyde (PFA) diluted in PBS for 1 h. After dehydrating sections by dipping slides serially in 50, 70, 100% ethanol, a hydrophobic barrier was drawn around each tissue section. Sections were treated with Protease 4 for 30 min at room temperature and rinsed with PBS for 2 min, and then incubated with RNAscope probes at 40°C for 2 h using the HybEZ Hybridization System (Advanced Cell Diagnostics). Excessive probes were removed by rinsing sections twice with wash buffer for 2 min, selective signal amplification was achieved by serial incubation of Amp 1–4 reagents. After the final wash, tissue sections were counterstained with DAPI and sealed with a coverslip using ProLong Diamond Antifade reagent (ThermoFisher Scientific; catalog no. P36970), and then stored at 4°C until imaged. All probes used in this study were purchased from Advanced Cell Diagnostics [human CRH (catalog no. 475211), SLC17A7 (catalog no. 415611), GAD1 (catalog no. 404031), PVALB (catalog no. 422181), SST (catalog no. 310591), VIP (catalog no. 452751)].

### Wide-Field Fluorescence Microscopy

Slides were viewed and analyzed using an Olympus IX83 microscope (Richmond Hill, ON, Canada) equipped with ProScan III XYZ motorized stage with linear XYZ encoders (Prior Scientific, Rockland, MA, USA). Images were captured using fluorescence microscopy and a Hamamatsu ORCA-Flash4.0 V2 digital CMOS camera (Bridgewater, NJ, USA). The hardware was controlled by SlideBook 6 (Intelligent Imaging Innovations, Inc., Denver, CO, USA), which was also used for post-acquisition processing.

All 3-D image stacks were acquired over the entire thickness of the tissue section using optimal exposure settings. Differences in exposures were normalized during image processing and each fluorescent channel was deconvolved using the AutoQuant adaptive blind deconvolution algorithm using Slidebook. Lipofuscin autofluorescence was detected as described previously (31).

### Characterization of Cellular Identity of CRH+ Cells

Cells in the sgACC of human controls were labeled using FISH technique with probes targeting CRH and markers of different populations of neurons (SLC17A7, GAD1, PVALB, SST, VIP). Twenty randomly sampled 3-D image stacks (~333 × 333 μm 2-D images sequentially captured at intervals separated by 0.75 μm in the z-dimension) were acquired within the gray matter using 20 × 0.75 NA objective. Cells containing ≥ 10 mRNA grain clusters were considered to express corresponding gene and colocalization patterns of CRH and each neuronal marker were analyzed.

To estimate the laminar distribution of CRH+ cells, the laminar position of the imaging site was determined based on the relative cortical depth, and then the average density of CRH+ cells in single image stacks (~333 × 333 μm) were multiplied with proportion of each layer in sgACC (L1: 12.4%, L2: 9.5%, L3: 30.4%, L5: 17.8%, L6: 30.0%) (32). The sgACC is agranular and does not contain a layer 4.

### Changes in CRH+ Cell Density and Cellular Expression of CRH mRNA

To investigate disease-dependent changes in cellular CRH mRNA level and cell density, three to four cortical traverses (1 mm wide by 2–4 mm depth) extending from the pial surface to the white matter were sampled for each subject. Each traverse was divided with a sampling grid of 125 × 125 μm. After defining layers based on relative cortical depth (L1: pia-12.4% depth of gray matter, L2: 12.5–21.9%, L3: 22–52.3%, L5: 52.4–70.1%, L6: 70.2-white matter) (32), 3-D image stacks (~111 × 111 μm images separated by 0.25 μm) were collected using a 60 × 0.40 NA super corrected oil immersion objective from sampling boxes in the middle of each layer. Thirty images were taken from each layer (150 images/subject).

Cell boundaries were defined manually based on distribution of CRH mRNA grains and DAPI. CRH mRNA grains were counted using FISH-quant, a Matlab toolbox for automated analysis of 3-D FISH images (33). mRNA grains of SLC17A7 or GAD1 overlaid on the CRH+ cell soma were used to distinguish CRH+ excitatory or inhibitory cells. Compared to the images taken using the 20X objectives in which mRNA grains looked cluster-like structures, images collected with the 60X objectives presented single mRNA grains easily distinguishable from others. Therefore, cells containing >20 grains were considered to be CRH+ neurons as assessed in a previous study (31).

### Cell Collection and RNAseq

For cell collection, 12 μm-thick fresh-frozen sgACC sections were thaw-mounted onto polyethylene naphthalate-membrane slides (Leica Microsystems, Concord, ON, Canada; catalog no. 11600289) and stored at −80°C. CRH-expressing inhibitory interneurons were visualized using FISH labeling with RNAScope Multiplex Fluorescent V2 Assay (Advanced Cell Diagnostics; catalog no. 323100) with some modification. On the day of experiment, slides were taken from −80°C and immediately immersed in chilled 4% PFA diluted in PBS for 5 min. Sections were dehydrated by dipping slides serially in 50, 70, 100% ethanol and a hydrophobic barrier was drawn around each tissue section. Sections were treated with hydrogen peroxide for 7 min to inactivate endogenous peroxidase and further treated with Protease 3 for 7 min at room temperature. After PBS wash, human CRH probe was applied on the section and incubated at 40°C for 1 h. Excessive probes were removed by rinsing sections twice with wash buffer for several seconds and selective signal amplification was achieved by sequential incubation of Amp 1–3, HRP-C1, TSA Plus Fluorescein (PerkinElmer; catalog no. NEL741E001KT) and HRP blocker. After the final wash, tissue sections were counterstained with Neurotrace red (Thermo Fisher Scientific; catalog no. N21482) and dehydrated with 100% ethanol. ~150 cells were collected from layer 1–3 of gray matter per each subject using a LMD7 system (Leica Microsystems) and processed for RNA extraction using Picopure RNA isolation kit (Thermo Fisher Scientific; catalog no. KIT0204).

Sequencing libraries are prepared using the SMARTer Stranded Total RNA-Seq Kit Pico Input Mammalian (Clontech, Mountain View, CA, USA; catalog no. 634413). cDNA fragment size distribution of each library was determined with Bioanalyzer (Agilent Technologies, Santa Clara, CA, USA) using high sensitivity DNA kit (Agilent Technologies; catalog no. 5067-4626) to check the presence of adapter dimers and gDNA contamination. Libraries were pooled and sequenced using Illumina MiSeq system (Illumina, San Diego, CA, USA) for quality check (QC), and then Illumina NovaSeq 6000 sequencer at Donnelly Sequencing Center at the University of Toronto (ccbr.utoronto.ca/donnelly-sequencing-center).

### Gene Alignment and QC

Sequencing data was analyzed as previously described (34). In brief, 2 × 100 bp paired-end reads were aligned to GRCh38 human reference genome (ftp.ensembl.org/pub/release-86/fasta/homo_sapiens/dna/) using HiSat2 (35) and Genomic-Alignments (36). After aligning genes to exons, noise was corrected by removing low expressing genes if 1) they had <10 reads and 2) were not detected in more than two thirds of samples. Among total reads obtained per subject (115,354,986 in average), 79.4 and 14.4% were aligned to genome and exon, respectively. In sum, 15,472 genes were analyzed in this study.

### Biological Function Analysis

Group differences in individual transcript expression were examined using DESeq2 (37). To control for multiple comparisons, we performed false discovery rate (FDR) correction using the *fdrtool* package. Differential expression significance was set at 25% FDR rate, and pathway enrichment significance was set at *p* < 0.05. To identify biological pathways affected by MDD, Gene Set Enrichment Analysis (GSEA) (38) was performed using Wald statistic-ranked gene lists and EnrichmentMap gene-set database (39) with default GSEA parameters, permuted 10,000 times. Using a Cytoscape plugin, EnrichmentMap (39), mutually-overlapping gene-sets were clustered together and significantly enriched gene sets were visualized based on degree of shared genes, calculated by a 50:50 ratio of the Jaccard similarity coefficient [size of (A intersect B)/size of (A union B)] and Overlap coefficient {size of (A intersect B)/size of [minimum (A,B)]}. Leading-edge genes (i.e., those driving the enrichment signal) were identified by finding genes which (1) were shared amongst a majority (>50%) of gene-sets in each cluster, and (2) passed significance thresholds in DE analysis.

### Gene Co-expression Network Analysis

We constructed gene networks with genes positively correlated to CRH to identify biological pathways relevant to CRH+ interneurons in two data sets. First, to identify cell-intrinsic changes, we calculated Pearson's correlation values between expression of CRH and 15,471 genes detected by CRH+ interneuron-specific RNAseq. Two hundred and ninety-two genes showed significant positive correlations with CRH expression and were further analyzed with Cytoscape (40) (version 3.8.0) with GeneMANIA plugin (41). The gene set was extended using the default settings (20 max resultant genes, 10 max resultant attributes, automatic network weighing) and all attributes. The analysis consisted of genes for which gene symbols were recognized by the GeneMANIA app. As a result, 8 genes were excluded (AC005747.1, AC011448.1, CNMD, ECPAS, H4C4, LRATD2, SELENOK, SLC35E2A). Second, to identify microcircuit-wide functional changes, a distinct CRH co-expression network was constructed using transcriptomic data from bulk tissue [anterior cingulate cortices of male subjects, *n* = 16/group; refer to (42) for cohort information]. One hundred and ninety unique genes were selected based on Pearson's correlation value to CRH level in control group (*r* > 0.55, *p* < 0.0254) and further examined with ClueGo, a Cytoscape plugin (v.2.5.7) (43) with GO Biological Process annotations (GO-BiologicalProcess-EBI-UniProt-GOA-ACAP-ARAP-08.05.2020).

## Results

### Cellular Identity of CRH (+) Neurons in Human Cortex

**Figure 1:**
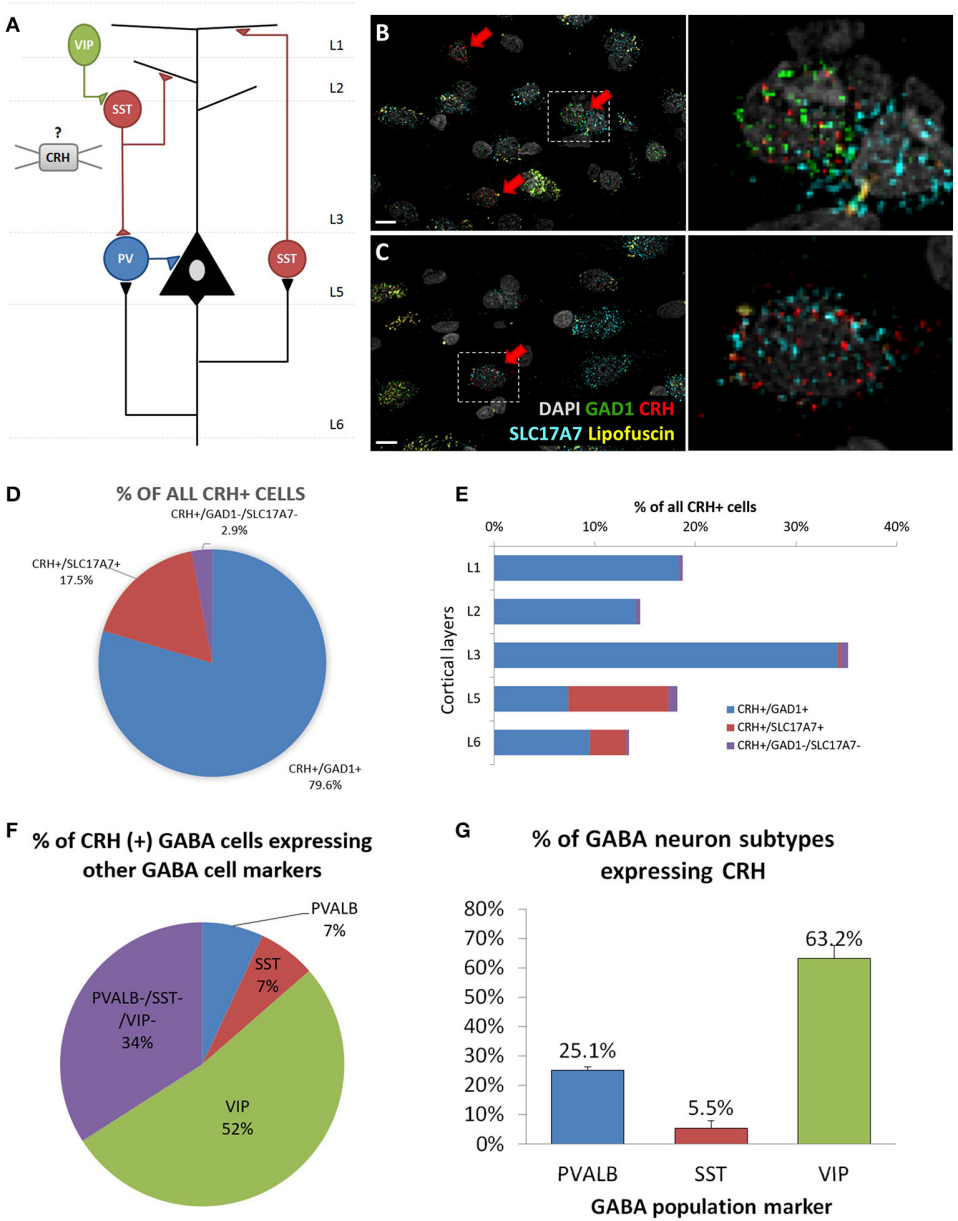
Cellular identity of CRH expressing neurons in human sgACC. **(A)** Schematic of the cortical microcircuit. **(B,C)** Images of human subgenual anterior cingulate cortex (sgACC) labeled for SLC17A7, GAD1 and CRH mRNAs and counterstained with DAPI (*n* = 8; scale bar: 10 μm). **(B)** Many GAD1+ GABAergic interneurons in layers 1–3 co-express CRH. **(C)** Some SLC17A7+ glutamatergic cells in layer 5 co-express CRH. **(D)** The majority of CRH expressing cells in human ACC are GABAergic. **(E)** CRH+ GABAergic interneurons are found across cortical layers while CRH+ glutamatergic neurons are concentrated in deep layers. **(F)** 66% of CRH+ neurons are overlapping with three major interneuron populations (52%: VIP, 7%: PVALB, 7%: SST) and 34% belongs to a separate subgroup of interneurons (*n* = 5). **(G)** Percentage of 3 major interneuron subgroups that express CRH.

To determine the cell type-specific expression of CRH in excitatory neurons and inhibitory interneurons of the sgACC (Figure 1A), we performed FISH with probes targeting CRH and markers of excitatory neurons (SLC17A7) and inhibitory interneurons (GAD1) (*n* = 6 subjects; Figures 1B,C). We found that CRH was primarily colocalized with GAD1; ~80% of CRH+ cells expressed GAD1 and 17.5% expressed SLC17A7. CRH+ GABAergic interneurons were found across all cortical layers (laminar distribution of CRH+/GAD1+ neurons: L1, 18.3%; L2, 14.1%; L3, 34.2%; L5, 7.4%; L6, 9.5%), while CRH+/SLC17A7+ (glutamatergic) neurons were concentrated in deep layers (L1 & 2, 0%; L3, 0.2%; L5, 9.9%; L6, 3.7%; Figure 1D).

To further characterize CRH+ GABAergic interneurons, we examined co-expression of CRH, GAD1 and markers of three non-overlapping subpopulation of interneurons (PVALB, SST, VIP; *n* = 5; Table 4, Supplementary Figure 1). We observed that CRH+ GABAergic interneurons in human sgACC are predominantly VIP+ (52%) and that a small proportion of the interneurons expresses SST (7%) or PVALB (7%); (Figure 1E). Notably, 34% of CRH+ interneurons expressed GAD1, but none of three interneuron markers. The percentages of SST-, PVALB- and VIP-expressing interneurons that expressed CRH were also calculated (Figure 1F). Similar to the high colocalization of VIP in CRH+ interneurons, 63.2% of VIP+, 25.1% of PVALB+ and 5.5% of SST+ GABAergic interneurons expressed CRH.

**Table 4:** Total number of SST-, VIP-, PVALB-expressing CRH+ neurons counted in human sgACC.

		**551**	**604**	**795**	**857**	**1,122**	**Mean**	**S.E**.
Section 1	GAD1+ cells	264	252	253	250	249	253.6	2.7
	CRH+ cells	73	84	66	109	75	81.4	7.5
	PVALB+ cells	15	30	23	26	9	20.6	3.8
	CRH+/GAD1+ cells	60	69	55	95	65	68.8	7
	PVALB+/GAD1+ cells	14	24	22	26	9	19	3.2
	PVALB+/CRH+/GAD1+ cells	4	6	5	7	2	4.8	0.9
Section 2	GAD1 (+) cells	265	235	240	316	261	263.4	14.4
	CRH (+) cells	84	65	58	114	59	76	10.6
	SST (+) cells	53	81	70	89	71	72.8	6.1
	CRH+/GAD1+ cells	71	60	56	96	50	66.6	8.1
	SST+/GAD1+ cells	50	81	68	87	65	70.2	6.5
	SST+/CRH+/GAD1+ cells	1	7	1	12	1	4.4	2.2
Section 3	GAD1 (+) cells	229	233	218	276	202	231.6	12.3
	CRH (+) cells	58	87	63	121	53	76.4	12.6
	VIP (+) cells	33	54	59	68	47	52.2	5.9
	CRH+/GAD1+ cells	48	69	57	93	50	63.4	8.3
	VIP+/GAD1+ cells	30	54	58	68	45	51	6.4
	VIP+/CRH+/GAD1+ cells	15	36	34	52	30	33.4	5.9

### Cellular Origin of Reduced CRH Expression in the sgACC in MDD

**Figure 2:**
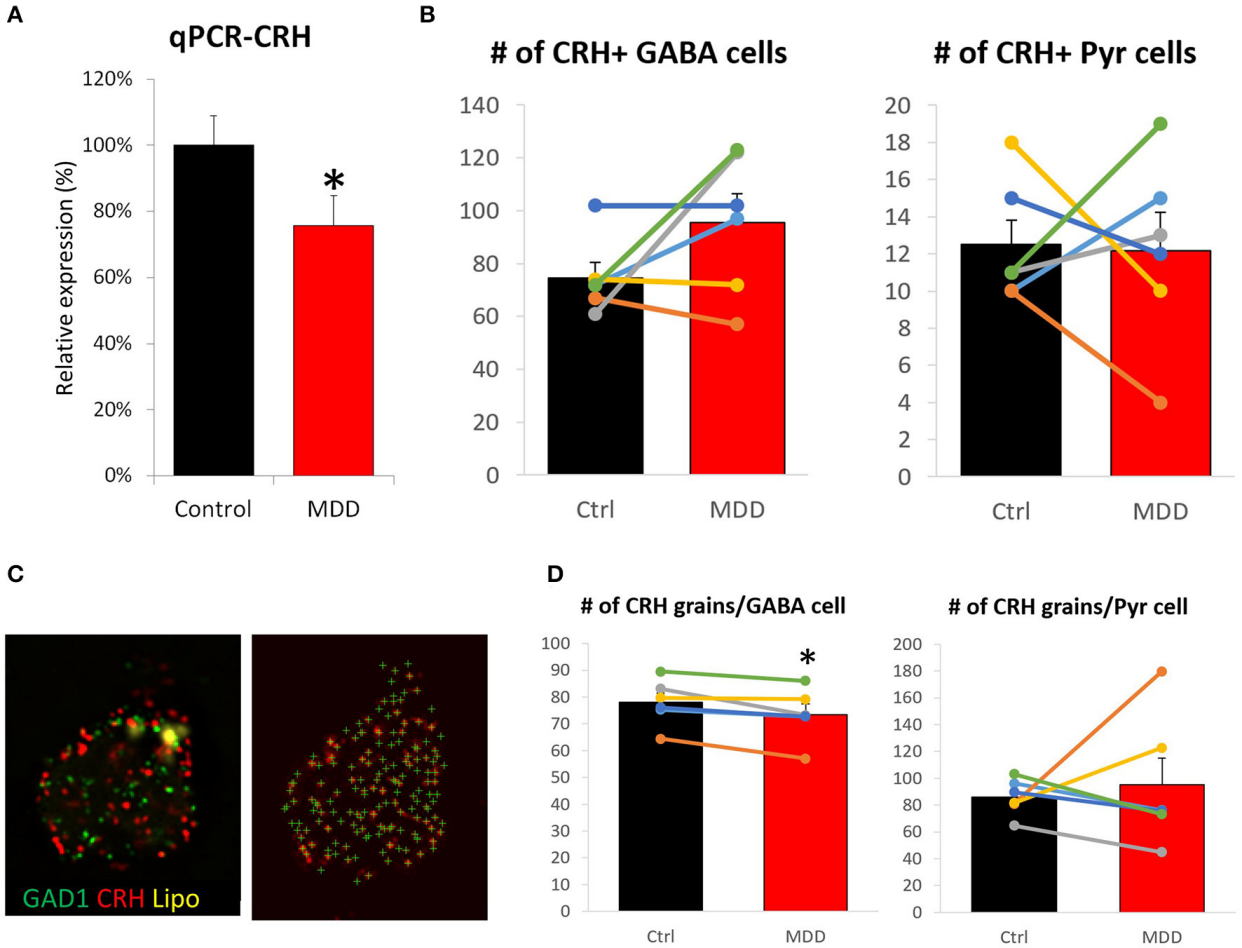
Differences in CRH level and cell density in the sgACC of MDD subjects. **(A)** CRH expression level is significantly reduced in the sgACC of MDD patients compared to control. **(B)** There was no group difference in CRH+ neuron density. **(C)** Right: Image of a human sgACC tissue section labeled for GAD1 (green), CRH (red). Autofluorescent lipofuscin shown in yellow. Left: Single channel image showing CRH grains detected by FISHquant. **(D)** Cellular CRH expression is reduced in GABAergic interneurons, but not in pyramidal neurons in the sgACC (*n* = 6/group; ^#^*p* < 0.1; *p < 0.05).

We previously reported lower CRH gene expression in corticolimbic areas of MDD patients (9). Yet, it has not been investigated whether cell loss and/or reduced cellular expression contributed to this change. A sub-cohort of 12 subjects (*n* = 6/group) was formed based on sample availability and significant CRH reduction in bulk tissue transcriptome data (9). qPCR results confirmed lower CRH expression in MDD (75.6 ± 9.1%, *p* = 0.018; Figure 2A) with high correlation to array data (*r* = 0.76, *p* = 0.003; Supplementary Figure 2A). Using the same subjects, we performed FISH to measure cell density and cellular CRH expression level. No significant differences in cell density were observed in either inhibitory interneurons (control: 74.7 ± 5.8, MDD: 95.5 ± 10.9, *p* = 0.15) or excitatory neurons (control: 12.5 ± 1.3, MDD: 12.2 ± 2.0, *p* = 0.90; Figure 2B). Detected number of CRH mRNA grains per cell (Figure 2C) showed significant reduction in GABAergic interneurons (control: 78.0 ± 3.5, MDD: 73.5 ± 3.9, *p* = 0.02; Figure 2D), but not in glutamatergic neurons (control: 86.0 ± 5.5, MDD: 95.4 ± 19.7, *p* = 0.67). Laminar analysis did not reveal significant group differences in cellular CRH expression level (Supplementary Figure 2B).

### Single Cell Type RNAseq Suggests Functional Hypoactivity and Reduced GABAergic Function of Cortical CRH+ Interneurons

**Figure 3:**
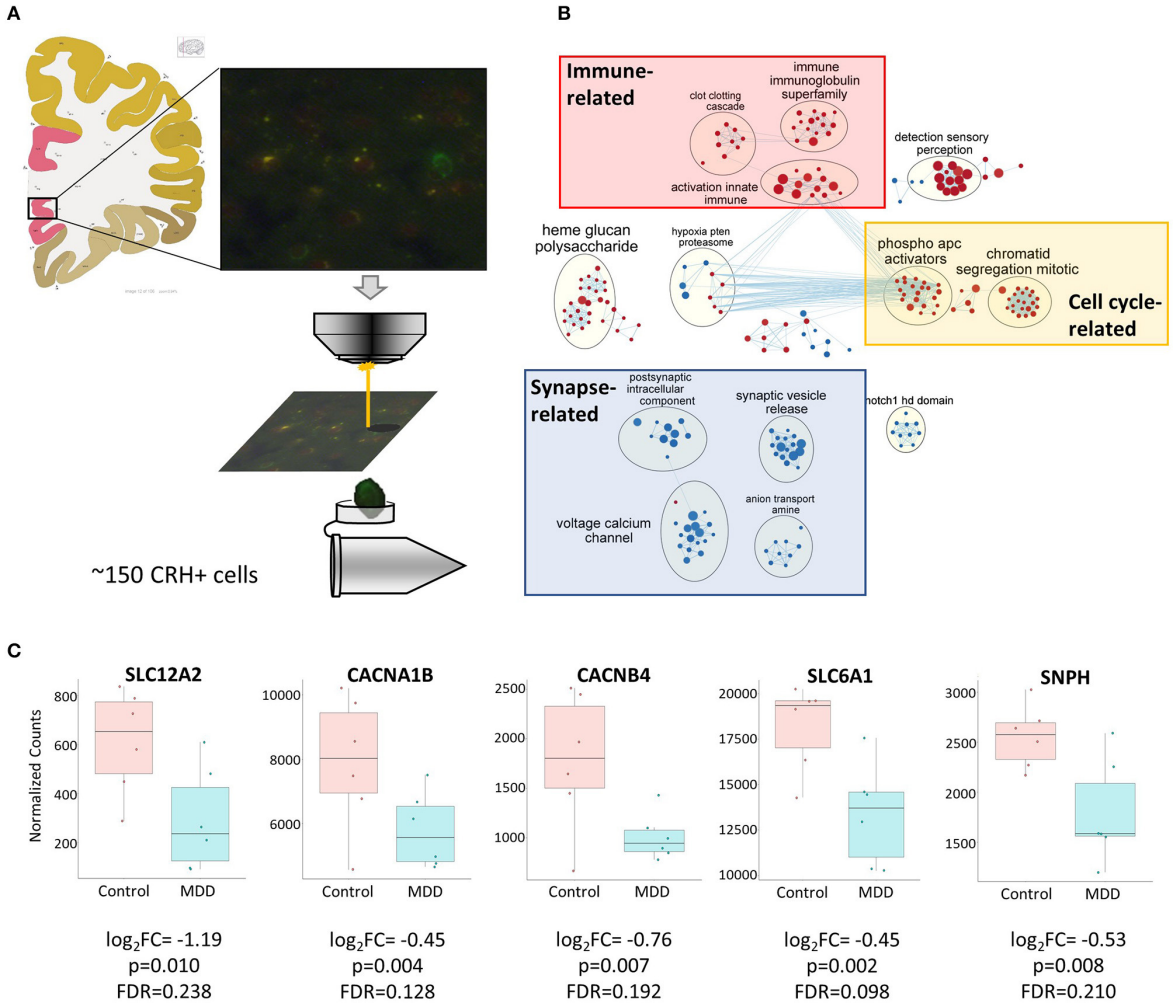
Biological functions associated with altered CRH+ GABAergic interneurons in MDD. **(A)** Scheme depicting CRH+ interneuron collection from human sgACC using laser capture microdissection. Image credit: Allen institute for brain science [https://atlas.brain-map.org/atlas?atlas=138322605#atlas=138322605&plate=102291577]. **(B)** Cell type-specific transcriptomic analysis revealed reduced synaptic function- and increased cell cycle-, immune-related function in sgACC CRH+ neurons of MDD patients (red: upregulated gene sets, blue: downregulated gene sets). **(C)** Reduced expression of synaptic related genes in the CRH+ neurons in the sgACC of MDD patients.

To assess disease-affected biological changes at the gene and pathway levels in cortical CRH+ GABAergic neurons, we performed RNAseq with CRH+ cells collected from superficial layers (L1–3) of sgACC (Figure 3A), which represents 79.8% of CRH+ GABAergic interneurons and 1.8% of CRH+ glutamatergic neurons (Figure 1D).

We identified 307 genes showing significant group differences [(168 upregulated, 139 downregulated; False discovery rate (FDR) <0.25). Next, we performed Gene Set Enrichment Analysis (GSEA)] with whole transcriptomic data to identify altered biological pathways in these interneurons. Five hundred and twenty-eight gene sets were significantly altered in MDD (267 upregulated, 261 downregulated; Supplementary Table 1). A pathway enrichment analysis was performed to summarize the GSEA output, which resulted in 3 altered biological themes: downregulated synapse-related (52 gene sets), upregulated cell cycle-related (42 gene sets) and immune-related (37 gene sets) pathways (Figure 3B). These changes were driven by 24 differentially-expressed genes including 9 synapse-, 5 immune-, 1 cell cycle-related pathway genes (Table 5). Impaired synaptic GABAergic function of CRH+ interneurons of MDD patients was implied from gene expression changes suggesting (1) cellular hyperpolarization (low SLC12A2 expression; also known as NKCC1), (2) less GABA release [downregulated voltage-gated calcium channels (CACNA1B, CACNB4)], less synaptic vesicle docking and fusion (decreased SNPH) and (3) less GABA reuptake (reduced expression of SLC6A1, also known as GAT1); (Figure 3C).

**Table 5:** Twenty-four differentially expressed leading edge genes.

	**Cluster**	**clustered GS**	**Gene symbol**	**Gene name**	**# of GS sharing**	**log2FoldChange**	***p*-value**	***q*-value**
Synapse related	Voltage calcium channel	17	CACNA1B	Calcium voltage-gated channel subunit alpha1 B	14	−0.45	0.004	0.128
			CACNB4	Calcium voltage-gated channel auxiliary subunit beta 4	14	−0.76	0.007	0.192
	Synaptic vesicle release	16	P2RX7	Purinergic receptor P2X 7	14	−1.27	0.004	0.128
			SNPH	Syntaphilin	14	−0.53	0.008	0.210
			CACNA1B	Calcium voltage-gated channel subunit alpha1 B	8	−0.45	0.004	0.128
	Postsynaptic intracellular component	10	PRR7	Proline rich 7, synaptic	7	−2.50	0.003	0.121
			ARF1	ADP ribosylation factor 1	5	−0.90	0.010	0.231
	Anion transport amine	9	ITGB1	Integrin subunit beta 1	9	−1.04	0.010	0.234
			P2RX7	Purinergic receptor P2X 7	8	−1.27	0.004	0.128
			SLC12A2	Solute carrier family 12 member 2 (NKCC1)	8	−1.19	0.010	0.238
			SLC6A1	Solute carrier family 6 member 1 (GAT-1)	8	−0.45	0.002	0.098
Immune related	Immune immunoglobulin superfamily	15	IL18R1	Interleukin 18 receptor 1	12	8.13	0.000	0.000
			IL1B	Interleukin 1 beta	12	6.70	0.001	0.063
			CD1E	CD1e molecule	8	7.48	0.001	0.034
	clot clotting cascade	9	CFHR4	Complement factor H related 4	7	5.14	0.011	0.248
			CPN1	Carboxypeptidase N subunit 1	7	5.21	0.007	0.189
			IL1B	Interleukin 1 beta	6	6.70	0.001	0.063
Cell cycle related	Chromatid segregation mitotic	21	TEX14	TESTIS expressed 14, intercellular bridge forming factor	21	3.31	0.011	0.248
etc.	Heme Glucan Polysaccharide	23	PHKG1	Phosphorylase kinase catalytic subunit gamma 1	13	3.58	0.004	0.128
			COX7C	Cytochrome c oxidase subunit 7C	12	0.97	0.010	0.238
			MT-CO2	Mitochondrially encoded cytochrome c oxidase II	12	0.41	0.007	0.187
	Detection sensory perception	13	OR9Q1	Olfactory receptor family 9 subfamily Q member 1	12	10.23	0.000	0.000
			OR2G6	Olfactory receptor family 2 subfamily G member 6	11	5.26	0.004	0.128
			OR51A4	Olfactory receptor family 51 subfamily A member 4	11	4.65	0.009	0.215
			TAS1R1	Taste 1 receptor member 1	7	6.00	0.008	0.215
	Notch1 hd domain	10	MIB2	Mindbomb E3 ubiquitin protein ligase 2	9	−1.03	0.004	0.128
			HEY2	Hes related family bHLH transcription factor with YRPW motif 2	8	−8.01	0.000	0.006

*GS, Gene Sets; Highlighted in pink, duplicated genes. Blue color indicates downregulated and pink color indicates upregulated*.

### Low Cortical CRH+ Interneuron Gene Expression Is Not Reflective of Excessive Glucocorticoid Feedback

**Figure 4:**
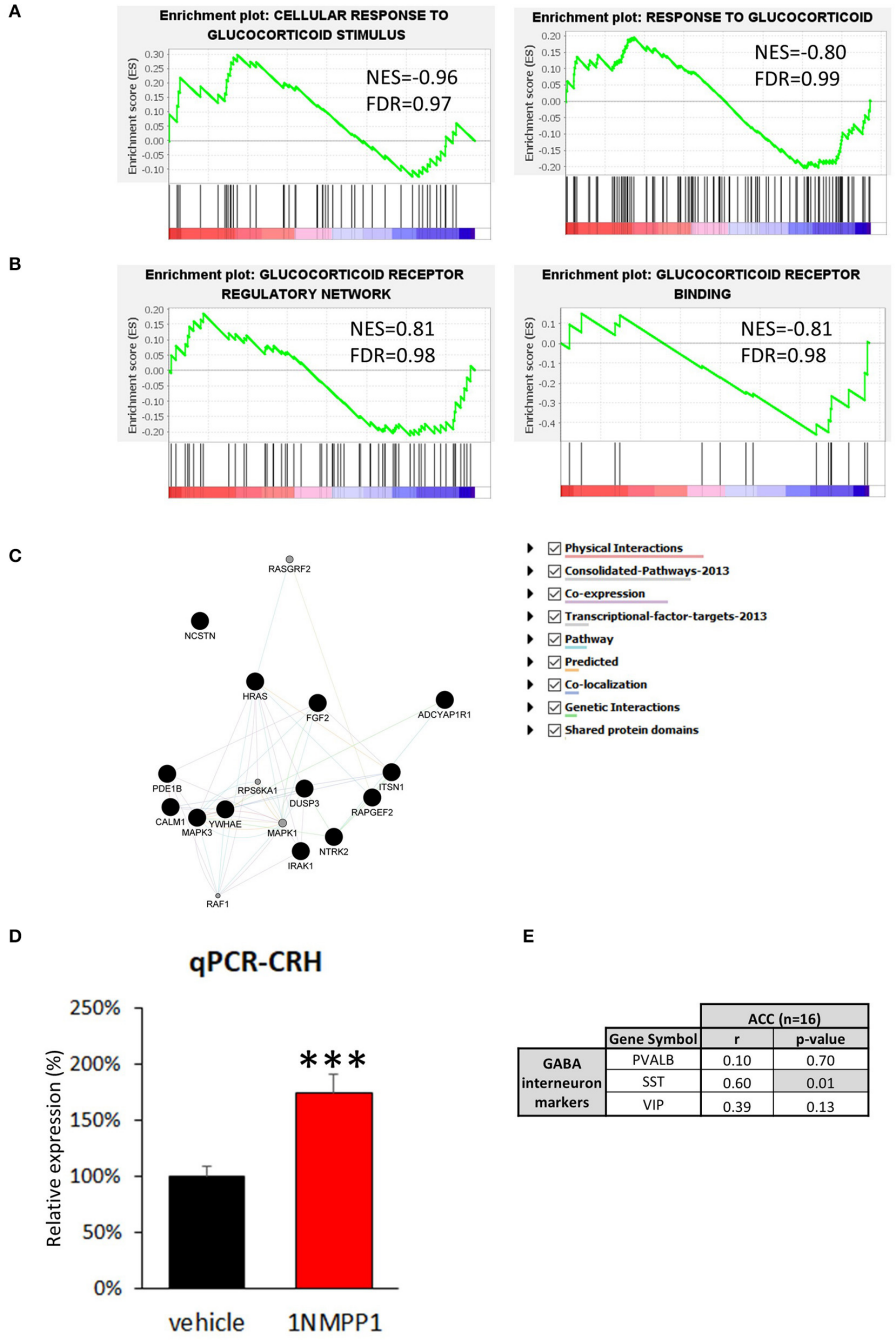
Possible upstream regulator of CRH. **(A,B)** GSEA results of four glucocorticoid-related signaling pathways. The enrichment plots of upregulated (upward curve) and downregulated (downward curve) genes in the relevant pathways do not differ from random distributions, hence they do not show significant differences in either direction. **(C)** Neurotrophin signaling pathway related genes found in bulk tissue CRH co-expression network (Black circle = genes originally included in CRH co-expression network, gray circle = genes added by GeneMANIA algorithm). **(D)** CRH expression level is significantly upregulated in the brains of NTRK2^F616A^ mice after 3 weeks of TrkB activity blockade (1NMPP1) (*n* = 4/group; ^#^*p* < 0.1; ***p < 0.001) compared to control (vehicle). **(E)** CRH expression shows high correlation to SST, and not to VIP and PVALB in bulk tissue dataset.

Low cortical CRH expression in MDD may result from a negative feedback due to enhanced glucocorticoid receptor signaling, since MDD patients tend to display higher level of peripheral cortisol (44). To test this hypothesis, we first examined differences in genes related to the response to glucocorticoids (Figure 4A). Neither of two relevant GSEA gene sets showed significant disease effect: response to glucocorticoid [GO: 0051384; normalized enrichment score (NES) = −0.80, FDR = 0.99], cellular response to glucocorticoid stimulus (GO: 0071385; NES = −0.96, FDR = 0.97). Additionally, we investigated 15 genes known to be directly downstream from glucocorticoid receptor activation identified from the Ingenuity knowledge base, an expert curated information on molecular network based on the literature, but we did not find significant changes (Supplementary Table 2).

Second, we determined differences in glucocorticoid signaling-associated genes (Figure 4B). Both glucocorticoid receptors, NR3C1 and NR3C2, were detected in our dataset but did not show significant MDD vs. control group difference [NR3C1: log2 fold change (LFC) = 0.78 ± 0.26, FDR = 0.99; NR3C2: LFC = 0.61 ± 0.29, FDR = 0.46]. Additionally, we examined three relevant gene sets and did not observe significant disease effect: glucocorticoid receptor regulatory network (*n* = 58 genes from Pathway interaction database NCI-Nature curated data; NES=0.81, FDR = 0.98) and binding partners of glucocorticoid receptor (GO: 0035259; NES = −0.81, FDR = 0.98). Glucocorticoid receptor signaling pathway-associated genes (GO: 0042921) were also analyzed and no significant change were detected (Supplementary Table 2). These results suggest that glucocorticoid signaling is not altered in CRH+ interneurons and that reduced CRH expression is unlikely due to an excessive CORT feedback mechanism.

### Reduced Cortical CRH Expression Is Indirectly Associated With Reduced Neurotrophic Factor Signaling

To identify biological pathways potentially mediating reduced cortical CRH expression, we analyzed genes highly correlated with CRH in CRH+ interneuron-specific transcriptome. We first identified genes showing significant positive correlation to CRH (*p* < 0.05, *n* = 292; Supplementary Table 3) and predicted function of our gene set using GeneMANIA. The extended gene network revealed that neurotrophin signaling pathway-related genes are the most enriched (GO: 0038179, *q*-value = 0.014), including the 17 following genes: ADCYAP1R1, CALM1, DUSP3, FGF2, HRAS, IRAK1, ITSN1, MAPK1, MAPK3, NCSTN, NTRK2, PDE1B, RAF1, RAPGEF2, RASGRF2, RPS6KA1, YWHAE (Figure 4C). Notably, expression of NTRK2 (also known as TRKB), the main BDNF receptor, showed high correlation to CRH level (Pearson's correlation *r* = 0.71, *p* = 0.01).

In view of the CRH/NTRK2 correlation, we next tested whether reduced CRH expression occurs downstream from reduced BDNF signaling [as frequently observed in MDD (24)]. For this, we performed qPCR with cDNA generated from cingulate cortices of mice with temporally blocked NTRK2 activity (1NMPP1 treated NTRK2^F616A^ mice) (28). Interestingly, CRH expression was upregulated after 1NMPP1 treatment (174.0 ± 16.5%, *p* = 0.004; Figure 4D). This finding shows that CRH expression is potentially negatively regulated by BDNF/NTRK2 activity, and suggests that the reduced CRH expression observed in MDD does not result from reduced BDNF signaling.

We next assessed gene expression changes related to CRH and NTRK2 levels in the brains of subjects with MDD. For this, we first performed a gene co-expression analysis with transcriptomic data from bulk ACC tissue of control subjects (*n* = 16), to gain insights on correlated activity across various cell types. We analyzed 190 genes displayed positive correlation with CRH expression (Pearson's correlation, *r* > 0.55, *p* < 0.0254; Supplementary Table 4). Among these 190 CRH-coexpressed genes, 180 genes showed reduced correlation to CRH expression in MDD group compared to control, suggesting a disturbed CRH gene coexpression network (Supplementary Table 4). A ClueGO analysis showed that multicellular organismal response to stress and electron transport chain-related GO terms were highly enriched (Supplementary Table 5), with SST significantly and positively correlated with CRH (SST: *r* = 0.60, *p* = 0.01; VIP: *r* = 0.39, *p* = 0.13; PVALB: *r* = 0.10, *p* = 0.70; Figure 4E), together suggesting potential contributions to CRH interneuron pathology from cortical cell microcircuit-wide changes.

## Discussion

Recent animal and human studies implicate CRH and CRH+ cells in the stress response and trajectory of MDD across episode and remission phases (15, 16, 45), however, the molecular characteristics and pathological features of cortical CRH+ cells in the human brain are largely unknown. Here, we report that CRH is mostly expressed in GABAergic interneurons and that it overlaps with the major classes of interneurons in the human ACC, specifically VIP+ interneurons (Figure 1). To our knowledge, this study represents the first anatomical characterization of CRH expression in multiple GABAergic interneuron types in the human ACC. Expanding from our previous findings of reduced CRH expression in the brain of MDD patients (9), we show that CRH downregulation reflects reduced cellular expression, rather than a difference in cell density (Figure 2). Cell-specific transcriptomic analyses suggest that the GABAergic function of CRH+ interneurons is impaired in MDD (Figure 3). Finally, we aimed to identify possible biological pathways mediating MDD-associated dysfunction of CRH+ interneurons. To this end, we first tested our previous hypothesis that decreased CRH level might result from negative feedback on central CRH due to HPA axis hyperactivity, however, we did not find evidence of enhanced (or changed) glucocorticoid signaling in these MDD samples as determined by mRNA surveys (Figures 4A,B). Gene co-expression analysis suggested that dysfunction of CRH+ interneurons might result from low neurotrophic support, however, this was not supported by studies in mice with reduced NTRKB signaling (Figures 4C,D), and instead implied that CRH+ interneuron dysfunction may occur downstream from signaling pathway(s) that also independently controls neurotrophic activity. High correlation between expressions of CRH and SST suggest that CRH+ cell function may be tightly linked with the function of SST+ inhibitory interneurons in the cortical microcircuit (Figure 4E), knowing that this link may take multiple direct or indirect forms (46).

It has been reported that CRH+ cells represent a heterogeneous population of neurons. Rodent studies demonstrated that CRH is expressed in glutamatergic neurons in the paraventricular nucleus of hypothalamus (47), and in GABAergic interneurons in other brain regions such as the central nucleus of the amygdala (48, 49), bed nucleus of the stria terminalis (47), hippocampus (50), and neocortex (11, 15). Similar to the results from rodent cortex, we show that human sgACC CRH+ cells are mostly GABAergic interneurons and largely overlap with the major classes of GABAergic interneurons. We found that a substantial fraction of CRH+ inhibitory interneurons does not express detectable levels of markers of major cortical interneurons. This could be due to species differences; PVALB-, SST-, VIP-expressing interneurons markers may represent 80–100% of interneurons in rodent cortex (51), however, the proportion of calretinin-expressing neurons, which usually do not express PVALB, SST, or VIP, are markedly increased in the human cortex (52, 53). Additionally, human single cell transcriptomic dataset (https://celltypes.brain-map.org/rnaseq/human_m1_10x) suggests that they might belong to Lamp5+ cell clusters, which were identified as layer 1 NDNF+ interneurons in a previous mouse study (54).

Our findings suggest that MDD is associated with dysfunction of cortical CRH+ interneurons. In addition to GABAergic interneuron-specific CRH reduction, evidence of impaired GABA transmission was discovered in CRH+ interneuron-specific transcriptome. One interesting property of CRH+ interneurons is that they may have opposing functions; they can inhibit as well as excite their synaptic targets with GABA and CRH, respectively. It is well-documented that CRH activates prefrontal excitatory neurons and mediates depressive- and anxiety-like behaviors (55–57). Though the impact of GABAergic inhibitory functions of PFC CRH+ interneurons on affect regulation and stress response remains unexplored, reduced inhibitory function of CRH+ interneurons is consistent with previous observations of preferential disruption of GABA-related genes compared to excitatory-related genes in MDD (9, 22, 24, 58–60). Simultaneous reductions in inhibitory GABAergic and excitatory CRH functions can result in complex consequences. Mouse studies show that CRH receptors are expressed in pyramidal neurons, but not in interneurons in neocortex (55); therefore, the excitatory effect of CRH would be pyramidal neuron-specific. Considering that the majority of superficial CRH+ cells, which were collected by LCM in the current study, are VIP+ interneurons with disinhibitory effects on pyramidal neurons through SST+ interneurons (61), GABA and CRH secretion by superficial CRH+ interneurons would synergistically activate pyramidal neurons. Hence, simultaneous downregulation of GABAergic function and CRH expression is predicted to result in hypoactivity of pyramidal neurons in the short term. These reduced inhibitory function of CRH+ interneurons may initially increase activity of SST+ interneurons and increases activity-dependent gene expression, but chronic activation may cause endoplasmic reticulum stress due to excessive protein synthesis and compromised function of SST+ interneurons (62) and results in hyperactivity of pyramidal neurons, which may cause the increased metabolic activity of the sgACC that is observed in MDD patients (63). Follow-up neurophysiological studies should address how impaired CRH+ GABAergic interneuron function impacts the activity of local microcircuit, including pyramidal neurons and interneuron populations.

We also found evidence of upregulated immune response- and cell cycle-related gene expression in cortical CRH+ interneurons of MDD subjects. It is well-documented that MDD patients show elevated levels of inflammatory cytokines in blood, cerebral spinal fluid and brain (64–68). Interestingly, the causality is bidirectional: psychosocial stress can increase levels of inflammatory cytokines (69, 70) and 15–80% of subjects receiving interferon alpha (IFN-α) treatment develop MDD (66, 70–72). Inflammation can cause hyperactivity of HPA axis (73), however, in IFN-α-treated patients, depressive symptoms were related to changes in cytokines, but not to cortisol (74). Although inflammation and immune response-related genes are mainly expressed by endothelial and glial cells, the same genes are also expressed by cortical neurons in non-pathological conditions (75). A recent study showed that siRNA-mediated inhibition of IL-18 signaling in basolateral amygdala was sufficient to repress depressive-like behavior in mice (76). This result suggests that, not only circulating IL-18, which is significantly increased in MDD patients (67, 77), but also ones expressed in the brain promote depressive-like phenotypes. We did not investigate what induces inflammation-related genes in CRH+ interneurons, but it is reported that CRH inhibits hypoxia-induced DNA-binding activity of NF-kB, a transcription factor that regulates the expression of various proinflammatory cytokines (78). Therefore, we consider that reduced CRH signaling may induce expression of inflammation-related genes *via* NF-kB.

In our previous study, an immune function-related gene module associated with CRH+ cells showed the highest probabilistic association to MDD (16), suggesting a causal role of elevated immune response in CRH+ cells on the disease trajectory. It is unclear how these elevated immune response- and inflammation-related gene expression in CRH+ interneurons is associated with the pathophysiology of MDD. But, previous report showed that enhanced IL6 signaling increases excitation/inhibition ratio by changing expression levels of NKCC1 and KCC2 (79). While we did not find a significant change in IL6 mRNA level, IL1B can induce IL6 expression (80), therefore, IL1B released from CRH+ interneurons might increase IL6 expression in glial cells which results in hyperactivity of neighboring excitatory neurons. As the activation of GABA_A_ receptors exerts anti-inflammatory effects (81–83), GABA deficit in sgACC may exacerbate local inflammation, creating as a positive feedback loop.

Considering a large body of literature suggesting reduced neuroplasticity in the brains of MDD patients and stressed animals, it was surprising to find an upregulation of the cell cycle-related pathway in CRH+ interneurons. Note that most prior studies were not cell type-specific, therefore, changes in a rare neuronal type, such as CRH+ interneurons, might not have been detected. Another possible explanation is that it reflects gene expression changes in cells surrounding our target cells. Indeed, we observed minor off-target contamination, mainly from oligodendrocytes and astrocytes, in LCM-based single cell type RNAseq data sets (84).

It has been reported that patients with MDD display elevated plasma cortisol level (85), impaired feedback regulation of the HPA axis (86, 87), although this finding is not universal (88). Prolonged and/or repeated glucocorticoid exposure is sufficient to induce behavioral and neurobiological changes in animals, which mirror many of the core symptoms and neurobiological changes associated with MDD (89–91). The reported neurobiological changes in sgACC of MDD patients, such as brain atrophy (92), decreased neuronal size (93), and reduced neurotrophin activity (24), overall fit with the expected brain changes in the presence of excessive glucocorticoids. Nonetheless, we did not find evidence that enhanced glucocorticoid signaling mediates cortical CRH+ interneuron dysfunction. That is, glucocorticoid response genes were not affected in CRH+ interneurons of MDD patients. Our data demonstrate that expression of glucocorticoid signaling pathway-related genes remains intact in CRH+ interneurons in MDD, suggesting that the lack of association between CRH and glucocorticoid pathway is not due to disrupted glucocorticoid signaling. It is important to note our small sample size and that not all subjects in our cohort were experiencing depressive episode at time of death (Table 2). Therefore, we cannot exclude a possibility that elevated glucocorticoid signaling exerted a temporal impact initiating molecular alteration of CRH+ interneurons.

Gene co-expression network analysis is a useful tool to identify functionally connected gene sets (46). Here, the data suggest that low neurotrophic signaling might be associated with CRH dysfunction. NTRK2 expression was significantly correlated with CRH expression, and this close link between neurotrophic signaling and CRH was also observed in studies from other research groups (identified by GeneMANIA analysis) (94–98). However, our qPCR experiment in mice with temporally blocked NTRKB signaling showed that low CRH expression is not a downstream effect of downregulated BDNF/NTRK2 signaling. Instead, the high correlation between NTRK2 and CRH suggests that they share an upstream regulator which mediate reduced NTRK2 (24) and CRH expression in the sgACC of MDD subjects. As BDNF and NTRK2 play important role in neuronal maintenance and plasticity, identification of this upstream regulator will provide an important insight on molecular mechanism underlying brain atrophy and cell loss observed in the sgACC of MDD (92).

We also found high correlation between CRH and SST in bulk tissue gene expression data, suggesting two potential mechanisms. First, activities of CRH+ and SST+ interneurons are tightly linked, and altered activity of CRH+ interneurons (specifically CRH+/VIP+ cells) may adjust the function of CRH+ interneurons to maintain the excitation/inhibition balance, and vice versa, SST+ interneurons may affect CRH+ cell functions. SST+ interneurons are biologically vulnerable and can be affected by various factors including hyper-glucocorticoid signaling and stress (99). We previously showed that acute pharmacogenetic inhibition of SST+ interneurons increased anxiety-like behavior, and that chronic inhibition (3 weeks) showed an opposite effect (100), which suggests homeostatic rebalancing occurred. Second, an upstream regulator, which regulates neurotrophins, may mediate changes in both cell types. Indeed, we have shown that SST expression is closely linked to BDNF activity (22, 59, 62).

In summary, our study shows that cortical CRH+ GABAergic interneurons display transcriptomic changes consistent with reduced inhibitory function, which might be indirectly associated with low neurotrophic signaling and/or low inhibitory tone of the local cortical microcircuitry in MDD. These results provide additional supporting evidence for impaired GABAergic function in the cortex in MDD.

### Limitations

First of all, we investigated a small cohort, hence our results should be considered as exploratory and hypothesis-generating. The study was performed in male subjects only. It is well-known that there are sex differences in the prevalence and pathology of major depression. The prevalence of MDD is twice higher in women than in men (101–105); female patients also show higher recurrence rate, and report higher number of symptoms, greater severity and different symptomatology than male MDD subjects (105–108). These differences suggest that the molecular mechanisms underlying the pathophysiology of MDD may differ between men and women (109, 110). Follow-up studies should be performed with a larger cohort including both male and female subjects.

Second, only CRH+ GABAergic interneurons were analyzed. If CRH+ glutamatergic neurons were compared, we might be able to find cell-type differences, and mechanism underlying the selective vulnerability of inhibitory interneurons. Furthermore, CRH+ cells were collected only from superficial layers. Kubota et al. reported that cortical CRH+ neurons express VIP or SST in a layer-dependent manner (11). Although SST-, PV- and SLC17A7-expressing CRH+ cells consist minor subgroups of CRH neurons, it would be interesting to see how they differ from VIP+/CRH+ interneurons. Single cell RNAsequencing, as well as basic neuroscience studies determining their electrophysiological properties and synaptic targets, will further help in deciphering the cortical CRH+ cell diversity.

## Data Availability Statement

The datasets presented in this study can be found in online repositories. The names of the repository/repositories and accession number(s) can be found at: https://www.ncbi.nlm.nih.gov/geo/; GSE193417.

## Ethics Statement

All procedures were reviewed and approved by the University of Pittsburgh Institutional Animal Care and Use Committee.

## Author Contributions

ES and HO conceived and designed the project, analyzed the data, and wrote the manuscript. DL collected and processed human postmortem brains for gene expression analysis. HO performed molecular biology, tissue staining, and imaging. DN analyzed RNAseq data. All authors read and approved the final manuscript.

## Funding

This study was supported by a project grant from the Canadian Institute of Health Research (CIHR) PJT-153175.

## Conflict of Interest

ES is founder and Acting Chief Scientific Officer of Damona Pharmaceuticals formerly known as Alpha Cog Inc., a drug development company with small molecules in the pipeline for treatment of cognitive deficits across brain disorders and aging. DL currently receives investigator-initiated research support from Pfizer and Merck and in 2012–2014 served as a consultant in the areas of target identification and validation, and new compound development to Autifony, Bristol-Myers Squibb, Concert Pharmaceuticals, and Sunovion. The remaining authors declare that the research was conducted in the absence of any commercial or financial relationships that could be construed as a potential conflict of interest.

## Publisher's Note

All claims expressed in this article are solely those of the authors and do not necessarily represent those of their affiliated organizations, or those of the publisher, the editors and the reviewers. Any product that may be evaluated in this article, or claim that may be made by its manufacturer, is not guaranteed or endorsed by the publisher.
