# Supplementary material for: Lower Levels of GABAergic Function Markers in Corticotropin-Releasing Hormone-Expressing Neurons in the sgACC of Human Subjects With Depression
Source: Front Psychiatry. 2022 Feb 25;13:827972. doi: 10.3389/fpsyt.2022.827972 (PMC8913899; doi:10.3389/fpsyt.2022.827972)
Supplement: Supplementary file 2 [file Data_Sheet_1.DOCX]

Supplementary Material

**Lower levels of GABAergic function markers in Corticotropin-Releasing Hormone-expressing neurons in the sgACC of human subjects with depression**

Hyunjung Oh, Dwight Newton, David Lewis, Etienne Sibille

# Supplementary Figures and Tables List:

**Supplementary Figure 1.** CRH is expressed in 3 major interneuron populations.

**Supplementary Figure 2.**

**Supplementary Table S1.** Significant GSEA results. 267 upregulated, 261 downregulated gene sets were identified. (Excel File)

**Supplementary Table S2.** Expression changes in glucocorticoid-related genes (Excel File)

**Supplementary Table S3.** Genes significantly correlated with CRH expression in the CRH+ cell transcriptome data (Excel File)

**Supplementary Table S4.** Genes positively correlated with CRH in the anterior cingulate cortex (bulk tissue) of control subjects and MDD-associated changes in CRH correlation. (Excel File)

**Supplementary Table S5.** Sets of GO terms enriched in CRH-coexpressed genes in human anterior cingulate cortex. (Excel File)

**Supplementary Figure 1. CRH is expressed in 3 major interneuron populations.**


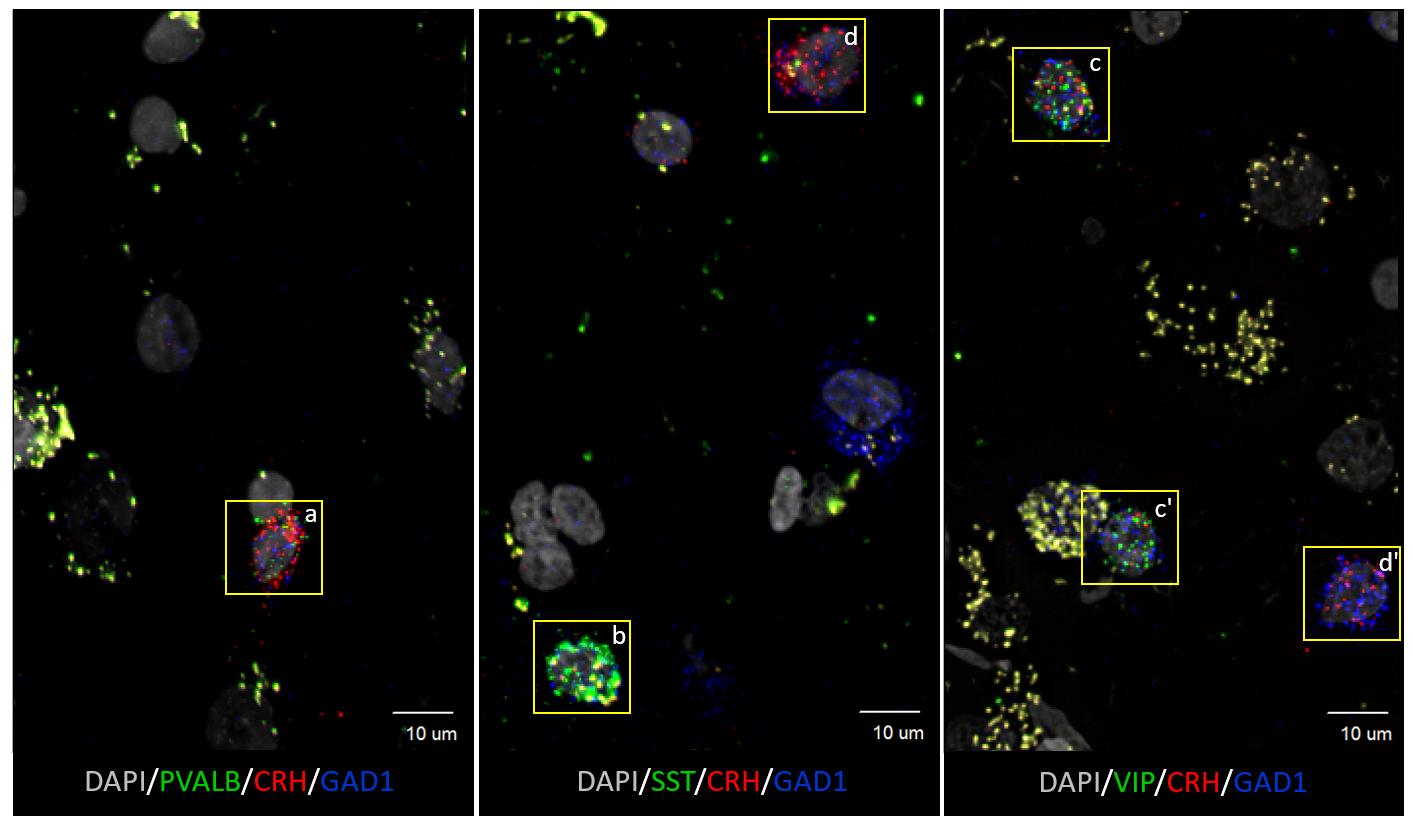


Representative images of human ACC labeled for GAD1, CRH, PVALB or SST or VIP mRNAs and counterstained with DAPI (n=5). CRH+ GABAergic interneurons coexpress PVALB (a), SST (b), VIP (c, c’) and none of three major interneuron markers (d, d’).

**Supplementary Figure 2.**


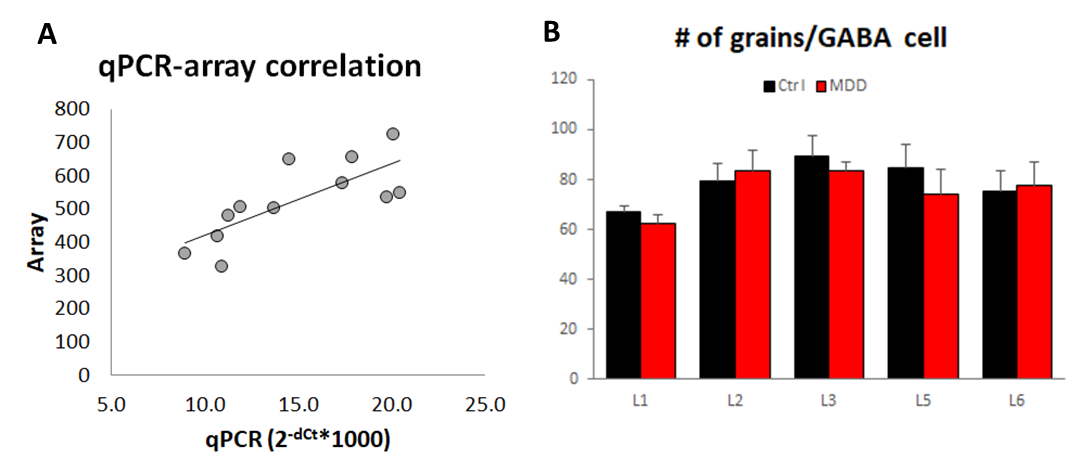


(**A**) High correlation between qPCR and array data (r=0.818, p=2.1X10-8). (**B**) Laminar changes of CRH mRNA grain numbers in GABAergic neurons
